# Supplementary material for: Stroke recovery phenotyping through network trajectory approaches and graph neural networks
Source: Brain Inform. 2022 Jun 19;9(1):13. doi: 10.1186/s40708-022-00160-w (PMC9206968; doi:10.1186/s40708-022-00160-w)
Supplement: Supplementary file 1 — Additional file 1: Appendix S1. Details of the ordinal (cumulative link) and Poisson (generalized linear) mixed-effect models. [file 40708_2022_160_MOESM1_ESM.pdf]

Appendix S1. Details of the ordinal (cumulative link) and Poisson (generalized linear) mixed-effect models.

##

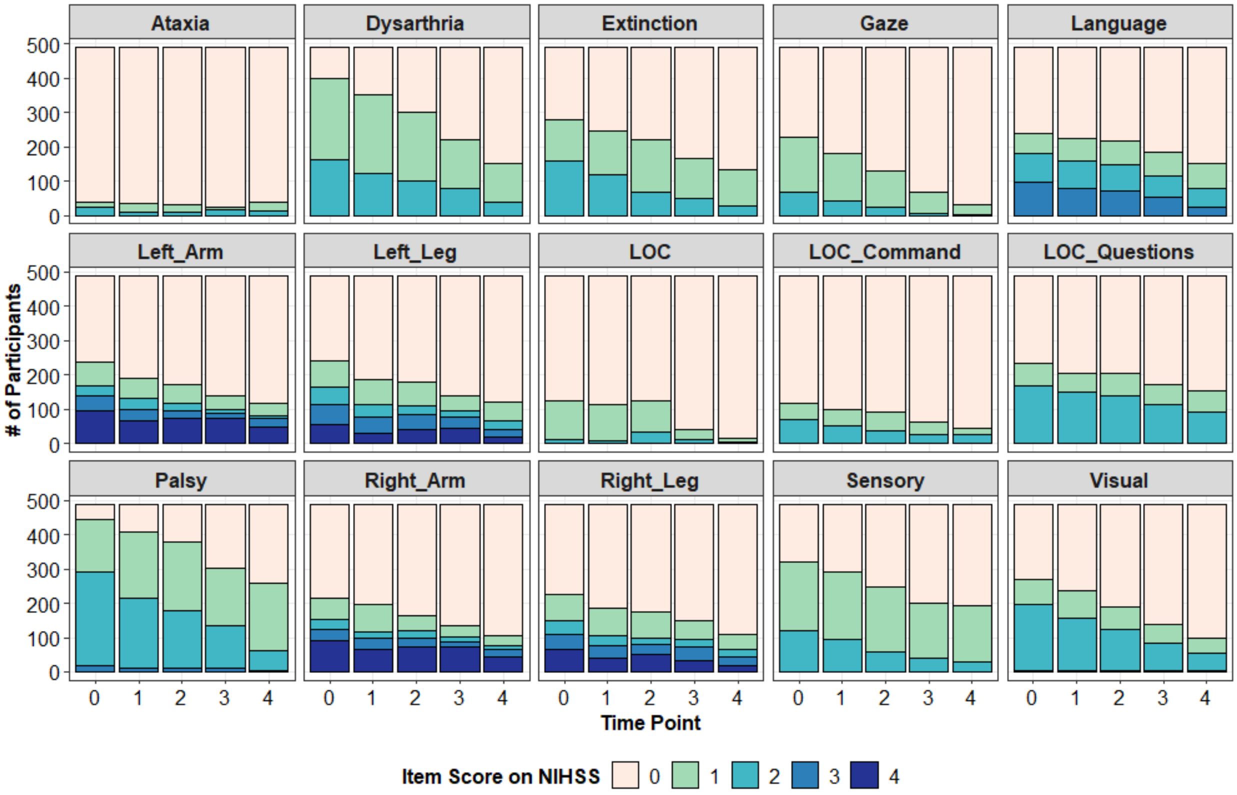

Figure S1. *Bar-plot showing the proportion of participants scoring at each level of different domains of the NIHSS at each time point (0=baseline, 1=~2-hrs, 2=~24-hrs, 3=7-10 days, 4=~90 days).*

Table S1. Fixed- and random-effects from the *ordinal cumulative link mixed-effects models for each domain of the NIHSS.*

	**Random-Effects**		**Fixed-Effects**		
**NIHSS Item**	σ_{Sub}	σ_{Time}	**Time**	**Group**	**Time x Group**
Ataxia	4.79	1.39	0.20 (0.11)	0.20 (0.39)	-0.08 (0.13)
LOC	1.14	0.19	**-0.43 (0.07)***	0.19 (0.16)	0.10 (0.06)
Dysarthria	2.04	0.43	**-0.76 (0.05)***	0.15 (0.21)	**0.13 (0.06)***
Extinction	1.99	0.41	**-0.38 (0.05)***	**0.51 (0.22)***	0.001 (0.061)
Gaze	2.15	0.55	**-0.68 (0.08)***	0.37 (0.25)	0.07 (0.08)
Language	3.84	0.62	**-0.39 (0.09)***	0.07 (0.47)	0.01 (0.09)
LOC Command	5.21	1.06	0.07 (0.09)	0.18 (0.26)	-0.08 (0.10)
LOC Questions	3.52	0.65	-0.010 (0.13)	0.74 (0.44)	-0.15 (0.10)
Left Arm	2.59	0.77	**-0.99 (0.11)***	0.19 (0.30)	**0.36 (0.11)***
Right Arm	2.99	0.72	**-0.65 (0.12)***	0.69 (0.49)	0.08 (0.10)
Left Leg	1.96	0.49	**-0.66 (0.07)***	**0.49 (0.22)***	0.13 (0.07)
Right Leg	2.47	0.62	**-0.55 (0.07)***	**0.74 (0.29)***	-0.03 (0.08)
Palsy	1.54	0.44	**-0.67 (0.05)***	0.22 (0.17)	**0.15 (0.06)***
Sensory	1.74	0.41	**-0.39 (0.05)***	**0.38 (0.19)***	0.06 (0.06)
Visual	2.26	0.56	-0.59 (0.06)	0.48 (0.24)	-0.04 (0.07)

Note that fixed-effects are listed as the estimate (SE). A 95% confidence interval can be approximated for each point estimate by taking 1.96 times the standard error. Ataxia, dysarthria, extinction, gaze, LOC command, LOC questions, and sensory ranged from 0-2; LOC, language, palsy, and visual ranged from 0-3; and left arm, right arm, left leg, and right leg ranged from 0-4. σ_{Sub} *= standard deviation of the random intercept for each subject;* σ_{Time} *= standard deviation of the random slope for the effect of time for each subject.*

N=489 for all models.

***** *Indicates a statistically significant effect, p<0.05.*

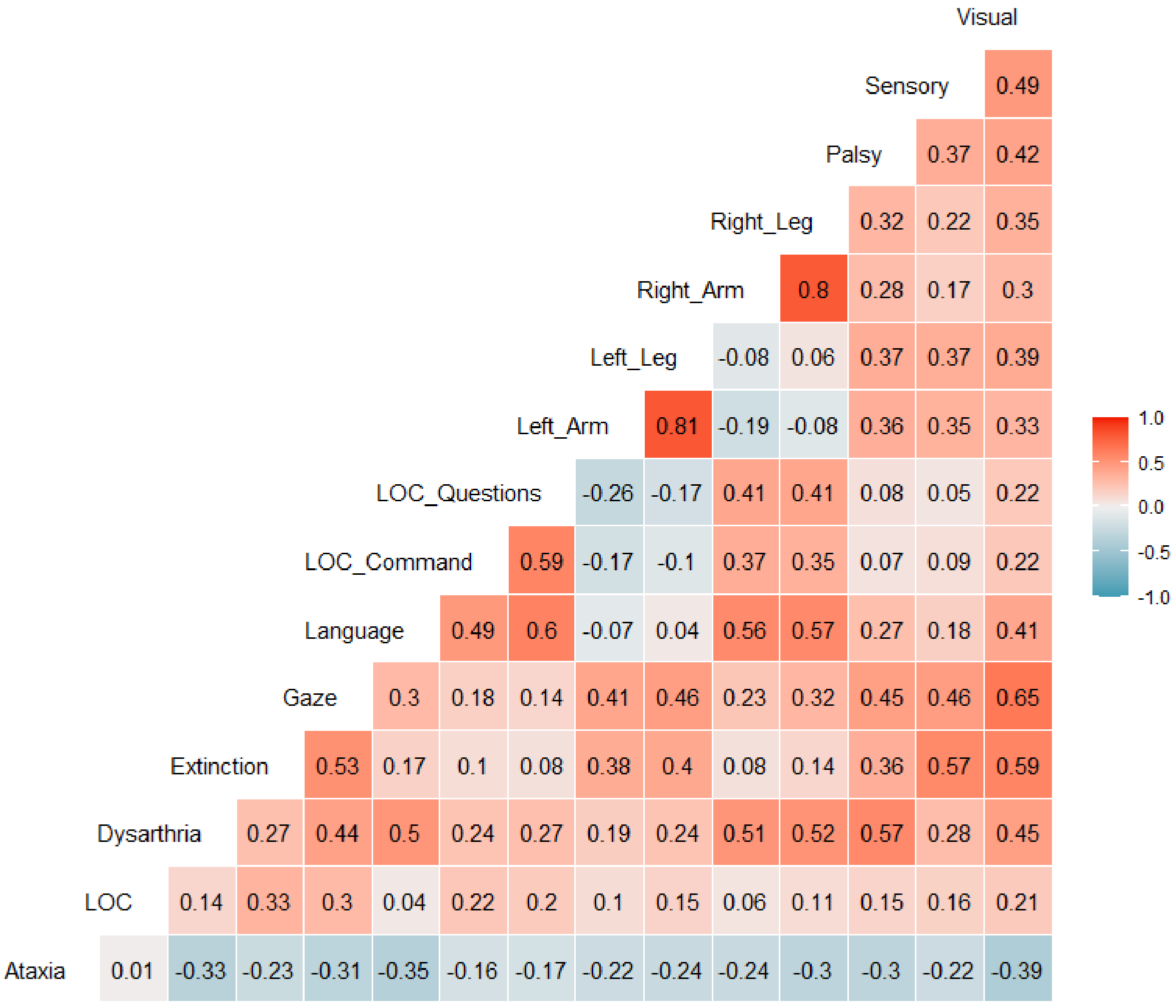

Figure S2. *Correlations between the random-slopes estimated for each subject from an* ***ordinal cumulative link mixed-effects model*** *for each domain of the NIHSS.*

Table S2. Fixed- and random-effects from *the Poisson generalized linear mixed-effects models for each domain of the NIHSS.*

	Random-Effects		Fixed-Effects		
**NIHSS Item**	σ_{Sub}	σ_{Time}	**Time**	**Group**	**Time x Group**
Ataxia	2.80	0.47	**0.51 (0.18)***	0.33 (0.47)	-0.12 (0.13)
LOC	0.97	0.18	**-0.58 (0.08)***	0.18 (0.16)	0.14 (0.07)
Dysarthria	0.35	0.17	**-0.39 (0.03)***	0.06 (0.07)	**0.81 (0.04)***
Extinction	0.71	0.12	**-0.37 (0.04)***	**0.22 (0.10)***	0.07 (0.04)
Gaze	0.80	0.21	**-0.75 (0.07)***	0.15 (0.13)	**0.14 (0.06)***
Language	1.44	0.17	**-0.32 (0.05)***	0.09 (0.16)	-0.001 (0.037)
LOC Command	1.74	0.24	**-0.43 (0.11)***	0.35 (0.23)	-0.08 (0.07)
LOC Questions	1.20	0.10	**-0.20 (0.04)***	0.23 (0.14)	-0.003 (0.037)
Left Arm	1.44	0.57	**-0.92 (0.09)***	0.21 (0.16)	**0.23 (0.07)***
Right Arm	1.61	0.44	**-0.68 (0.08)***	0.29 (0.18)	0.10 (0.06)
Left Leg	1.29	0.40	**-0.69 (0.12)***	0.31 (0.15)	**0.15 (0.06)***
Right Leg	1.44	0.37	**-0.55 (0.07)***	**0.36 (0.16)***	0.04 (0.06)
Palsy	0.19	0.11	**-0.28 (0.02)***	0.05 (0.06)	**0.08 (0.03)***
Sensory	0.49	0.10	**-0.28 (0.03)***	0.15 (0.09)	0.07 (0.04)
Visual	0.84	0.28	**-0.53 (0.05)***	0.17 (0.11)	0.03 (0.05)

Note that fixed-effects are listed as the estimate (SE). A 95% confidence interval can be approximated for each point estimate by taking 1.96 times the standard error. Ataxia, dysarthria, extinction, gaze, LOC command, LOC questions, and sensory ranged from 0-2; LOC, language, palsy, and visual ranged from 0-3; and left arm, right arm, left leg, and right leg ranged from 0-4. σ_{Sub} *= standard deviation of the random intercept for each subject;* σ_{Time} *= standard deviation of the random slope for the effect of time for each subject.*

N=489 for all models.

*** *Indicates a statistically significant effect, p<0.05.*

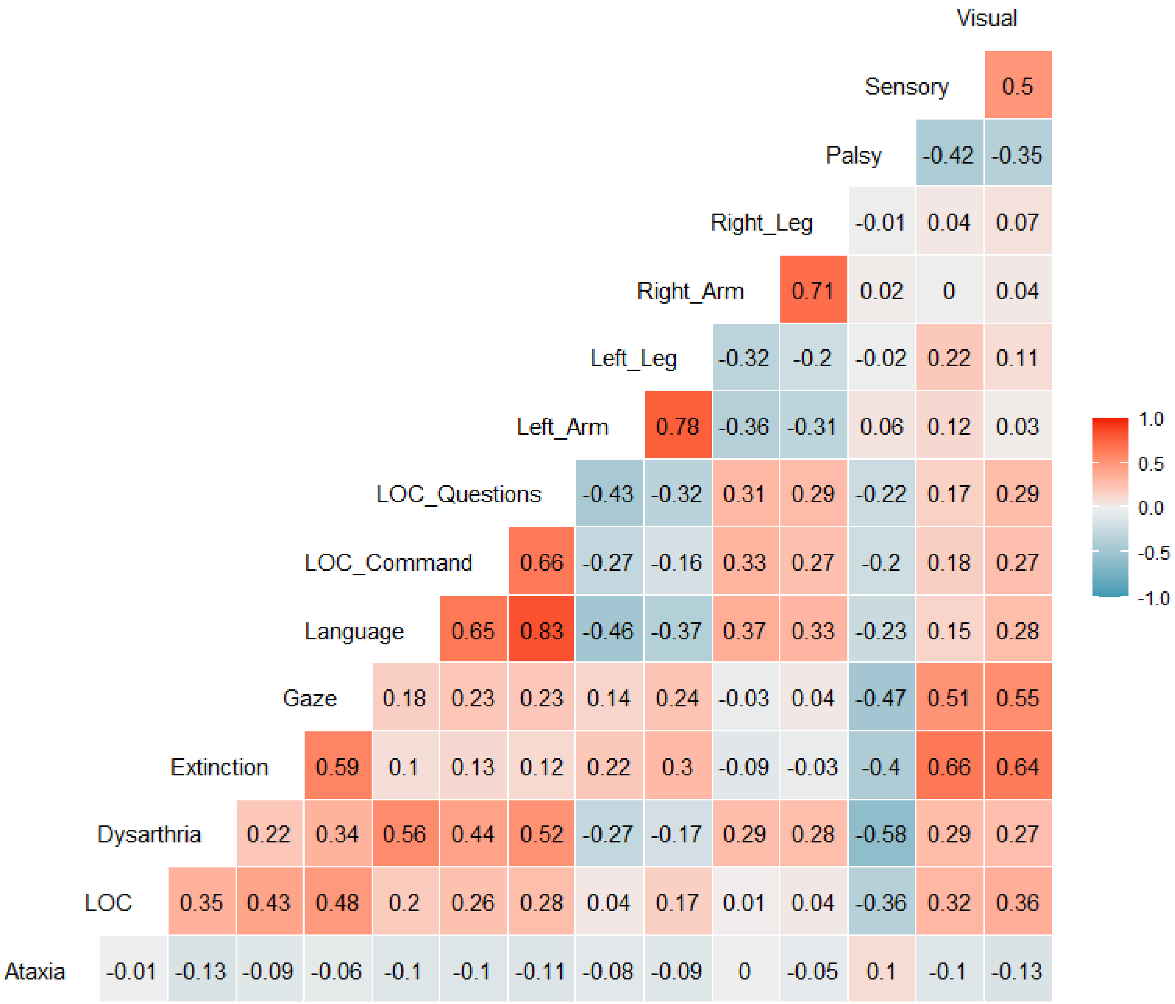

Figure S3. *Correlations between the random-slopes estimated for each subject from a* ***Poisson generalized linear mixed-effects*** *model for each domain of the NIHSS.*

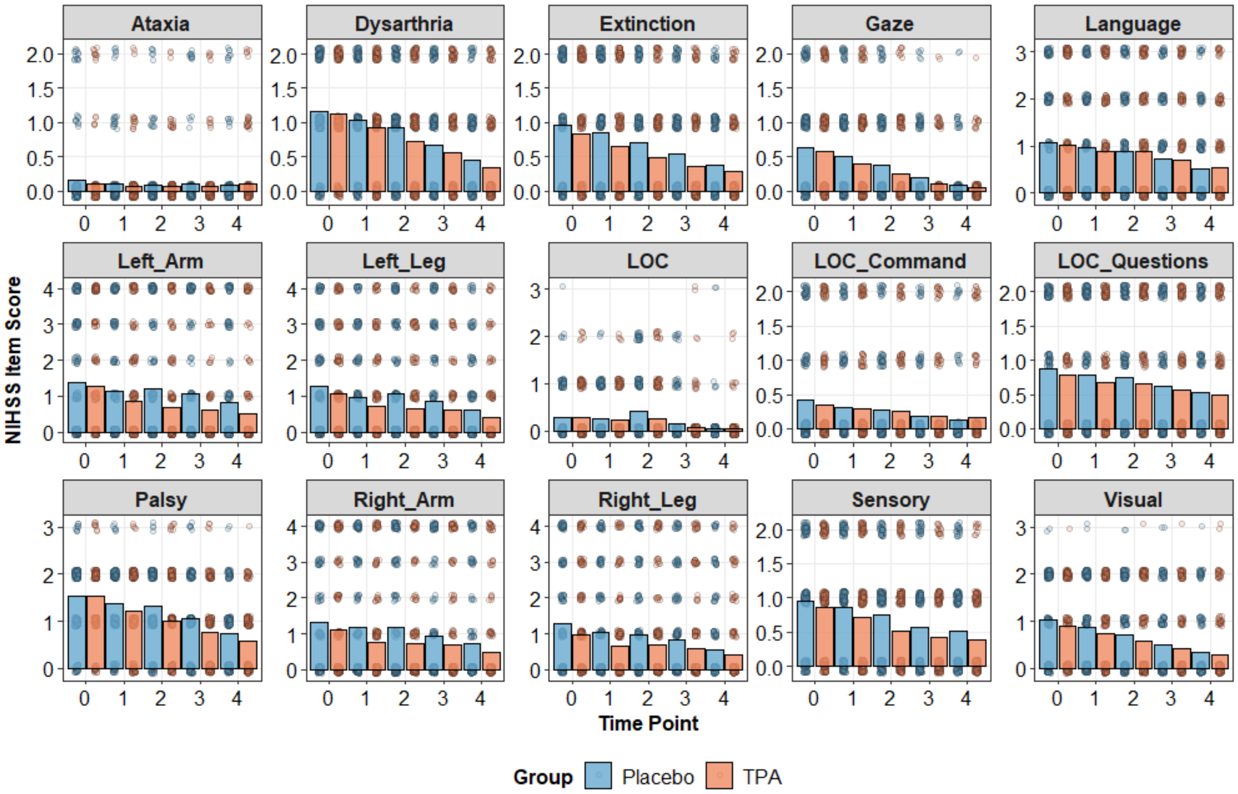

Figure S4. *Scatter plot showing the item scores for the different domains of the NIHSS as function of treatment group (TPA versus placebo) and time (0 = baseline, 1 = ~2-hrs, 2 = ~24-hrs, 3 = 7-10 days, 4 = ~90 days). Bars show the mean score for each group at each time; individual points are jittered to show overlapping data.*
